# Alcohol Impairs Predation Risk Response and Communication in Zebrafish

**DOI:** 10.1371/journal.pone.0075780

**Published:** 2013-10-07

**Authors:** Thiago Acosta Oliveira, Gessi Koakoski, Luiz Carlos Kreutz, Daiane Ferreira, João Gabriel Santos da Rosa, Murilo Sander de Abreu, Ana Cristina Vendrametto Giacomini, Ricardo Pimentel Oliveira, Michele Fagundes, Angelo Luis Piato, Rodrigo Egydio Barreto, Leonardo José Gil Barcellos

**Affiliations:** 1 Programa de Pós-Graduação em Farmacologia, Universidade Federal de Santa Maria (UFSM), Santa Maria, RS, Brazil; 2 Programa de Pós-Graduação em Bioexperimentação, Universidade de Passo Fundo (UPF), Passo Fundo, RS, Brazil; 3 Programa de Pós-Graduação em Ciências Ambientais, Universidade Comunitária da Região de Chapecó, Chapecó, SC, Brazil; 4 Department of Physiology, Bioscience Institute, Caunesp, Unesp, Botucau, SP, Brazil; Tulane University Medical School, United States of America

## Abstract

The effects of ethanol exposure on *Danio rerio* have been studied from the perspectives of developmental biology and behavior. However, little is known about the effects of ethanol on the prey-predator relationship and chemical communication of predation risk. Here, we showed that visual contact with a predator triggers stress axis activation in zebrafish. We also observed a typical stress response in zebrafish receiving water from these conspecifics, indicating that these fish chemically communicate predation risk. Our work is the first to demonstrate how alcohol effects this prey-predator interaction. We showed for the first time that alcohol exposure completely blocks stress axis activation in both fish seeing the predator and in fish that come in indirect contact with a predator by receiving water from these conspecifics. Together with other research results and with the translational relevance of this fish species, our data points to zebrafish as a promising animal model to study human alcoholism.

## Introduction

The effects of ethanol exposure on *Danio rerio* have been studied from developmental biology and behavioral perspectives [1]–[3]. However, little is known about the effects of ethanol on the prey-predator relationship and the chemical communication of predation risk.

It has been well documented that prey fish are able to recognize predation risk and communicate to conspecific fish through either chemical cues from epidermal club cells released as a result of skin injury (alarm substance) [4], [5] or disturbance substances released in the absence of a skin injury [6]–[8]. However, these studies focused on the antipredatory behavior triggered by alarm and disturbance cues. Studies focusing on the stress axis activation by chemical (alarm and/or disturbance) cues released from conspecifics that are in direct and/or indirect contact with a predator are scarce [9], [10]. The prey-predator relationship may trigger the stress axis in the prey fish [11] with either direct (in the presence of the predator) or indirect contact (visualization of the predator) [12], [13].

In predator–prey interactions, the early detection of the predator by the prey can be considered the first phase of the anti-predator response because it effectively allows the prey to avoid a direct contest with the predator. In fish, the early detection of predators (prior to physical contact) can be mediated by visual [14] and/or chemical [7]–[8] stimuli.

Previous research has demonstrated the effects of alcohol on several behavioral parameters [1], [2], and its effects are similar to those observed in fish exposed to anxiolytic drugs [15]. However, there is little information regarding the effects of waterborne alcohol on the stress axis activation of prey fish after visual contact with a predator or conspecifics that receive water from these fish seeing a predator.

## Materials and Methods

### Ethical Note

This study was approved by the Ethics Commission for Animal Use (CEUA) of the Universidade de Passo Fundo, UPF, Passo Fundo, RS, Brazil (Protocol#7/2013-CEUA), and met the guidelines of the Brazilian College for Animal Experimentation (COBEA; http://www.cobea.org.br).

### Animals

A population of 2200 mixed-sex, adult wild-type zebrafish (*Danio rerio*) from short-fin (SF) strain, were held under natural photoperiod (approximately 14 h light: 10 h dark). Water was maintained at 28.0±2.0°C, pH 7.0±0.6 units; dissolved oxygen at 6.8±0.4 mg L^−1^; total ammonia at <0.01 mg L^−1^; total hardness at 6 mg/L; and alkalinity at 22 mg L^−1^ of CaCO_3_.

### Experimental Design – General Information

Prior to each trial, groups of 12 fish for each treatment were acclimated in the experimental aquaria for three days, kept under the 14–10 h day/night cycle and fed three times a day with commercial flakes (TetraMin®, Tetra, Melle, Germany). Twenty-four hours before experimentation, fish were transferred to the experimental room. To avoid the influence of circadian rhythm on cortisol secretion, all samples were collected at the same time of day (11∶00 AM). The feeding of animals that were used as either predatory or non-predatory stimulus was suspended for 24 h before the start of the trial period to encourage possible predatory behavior.

**Figure 1:**
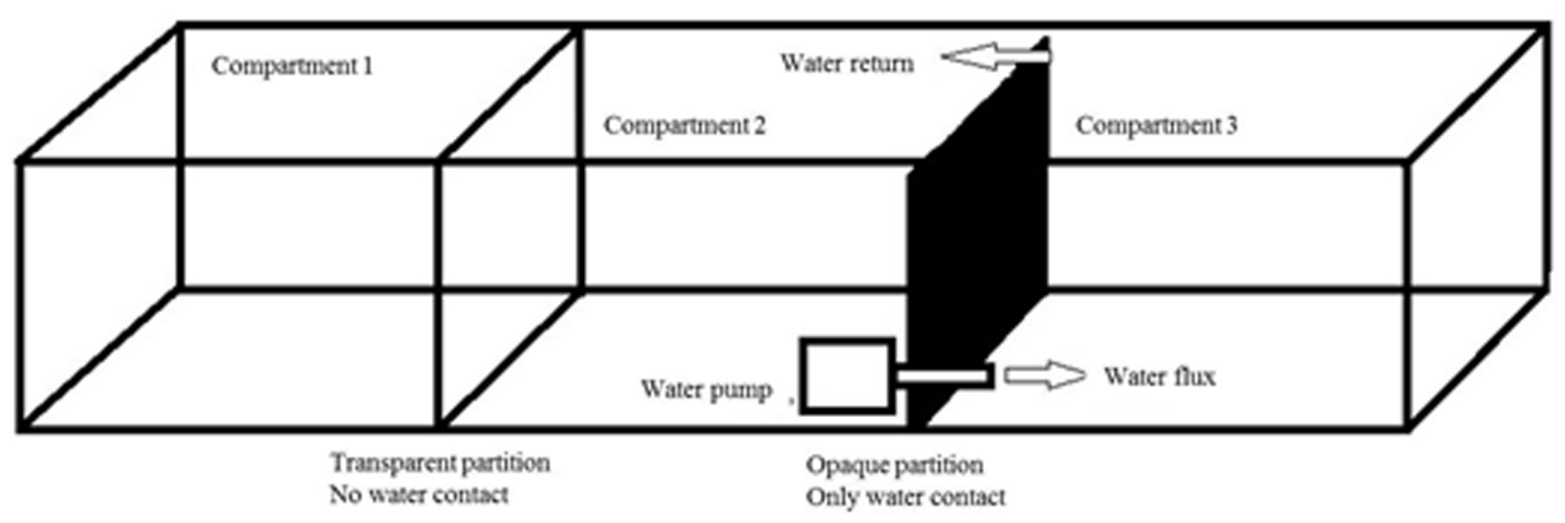
Schematic view of the experimental aquaria.

Large glass aquaria (120×40×40 cm) were divided into three compartments of the same size (Fig. 1). The glass partitions were fixed permanently in the experimental aquaria. The first and second compartments were divided by a transparent glass partition that permitted visual prey-predator contact. The second and third compartments were divided by an opaque partition that prevented visual prey-predator contact. Chemical communication was achieved with water circulation between the compartments through a submersible pump installed in a hole (1.5 cm in diameter) near the bottom. Water circulated continuously (approximately 3 L/min); the circulation efficiency was demonstrated by dispersion of water mixed with methylene blue from one compartment to the other. During each experimental trial, the aquaria were not cleaned, the water was not changed, and the fish were not fed to avoid undesirable chemical factors.

### Study 1 - Communication of Predation Risk in Fish Exposed to Alcohol

**Figure 2:**
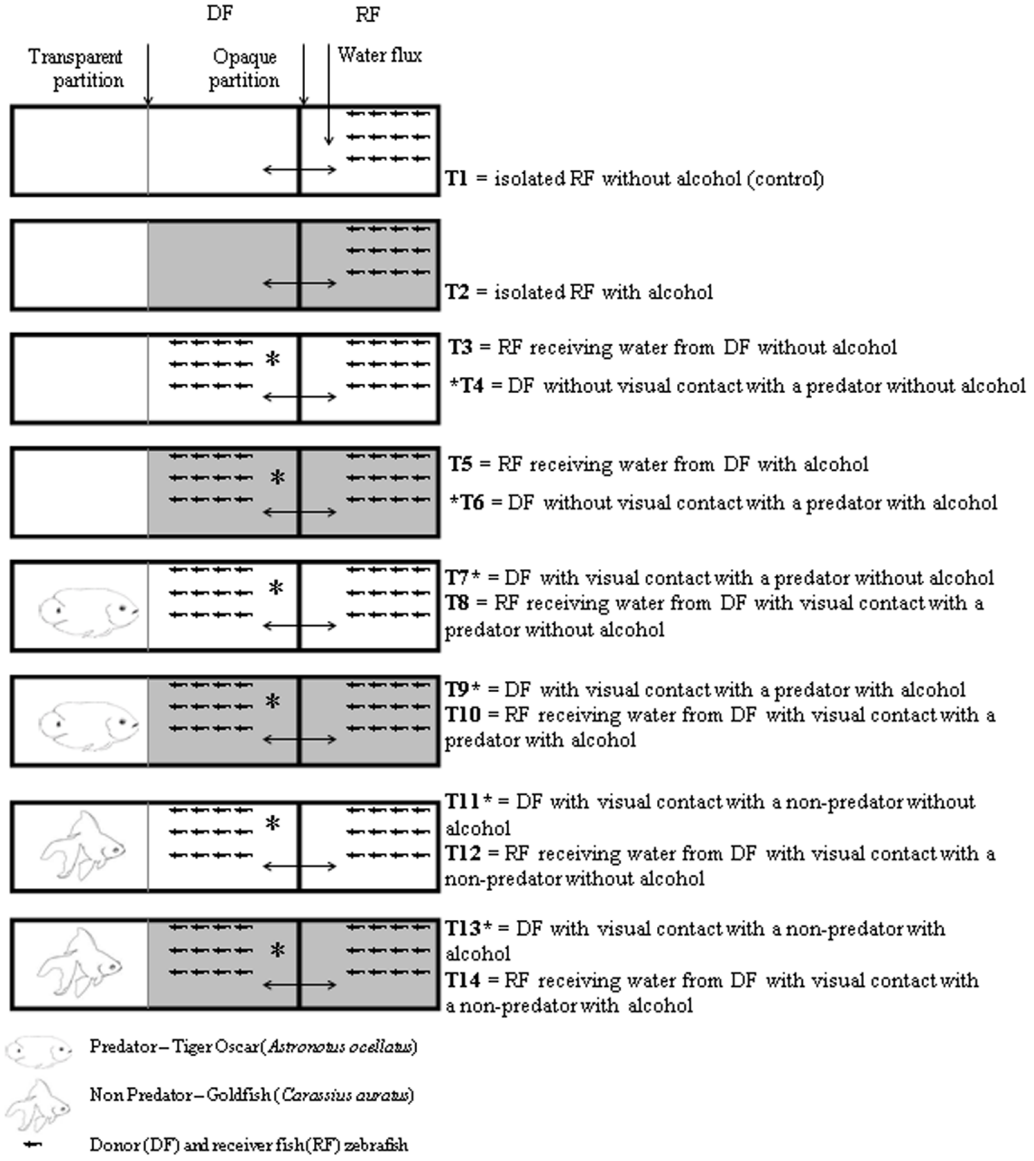
Schematic view of the experimental groups to study the effect of alcohol exposure on the communication of predation risk. White and gray represent treatments without or with alcohol, respectively.

We measured the effects of alcohol on stress response in a fish group subjected to visual contact with both a predatory and a non-predatory fish (stimuli fish, SF). After visual contact was established, the water from stimuli-exposed zebrafish (donor fish, DF) was transferred to conspecifics (receiver fish, RF). A total of 14 experimental groups were used to allow for repeated testing of three alcohol concentrations (0.25%, 0.50% and 1.00%) (Fig. 2).

Briefly, T1 and T2 testing only if the maintenance in the compartment 3 might cause stress in zebrafish with or without alcohol. T3 to T6 testing the presence of both DF and RF fish in the aquaria without SF in the presence or not of alcohol. The treatments T7 to T10 testing both DF and RF fish in the presence of predatory SF in the presence or not of alcohol. Groups T11 to T14 reproduces groups T7 to T10 but with a non-predatory SF.

Each zebrafish group (12 fish per group) were introduced into compartments 2 and 3 and habituated for at least three days. At the beginning of the experimental trial, fish of both compartments 2 and 3 were exposed to alcohol for 15 minutes (both the alcohol concentration and exposure time were based on previous studies [2]). The alcohol introduction was made in compartments 2 and 3 at the same moment using two glass pipettes. The water flux promoted by the pump rapid disperses and homogenizes the alcohol introduced. In non-exposed groups we repeat exactly the same procedure but using only water.

After treatment, DF groups were exposed to visual contact with the SF for 60 minutes. At the end of this period, both the DF and RF were sampled to whole-body cortisol analysis [9], [11]. Three replicates for each experimental trial were done.

The tiger Oscar (*Astronotus ocellatus*), a Cichlid fish with strong predatory behavior [16], was used as the predator for the SF, and goldfish (*Carassius auratus*) were used as the non-predator fish due to its peaceful and friendly temperament [17]. The SF fish were not exposed to alcohol and not used in any analyses. After each trial, they were returned to their original aquarium.

### Study 2 - Alcohol Effects on Stress Response

Because some studies have reported that alcohol may affect directly the stress response in animals and humans [18]–[24] and considering that the cortisol was used here as an indicator of chemical communication between DF and RF, we also evaluated the peak cortisol levels in zebrafish acutely exposed to physical stress and/or alcohol.

For this experiment, 144 zebrafish were distributed in 24 glass aquaria (30×30×30 cm, 27 L, 6 fish per aquarium). The control and stressed groups were either exposed to 0.50% alcohol for 15 minutes or untreated. Immediately after alcohol exposure, a standard acute stressor consisting of chasing fish with a net for two minutes was applied. Fifteen minutes after the stressor was removed, fish were sampled for whole-body cortisol analysis. The time periods used were established by Ramsay et al. [25], who determined the interval between the stressor and peak cortisol concentrations.

### Study 3 - Effects of a Submersible Pump

Because both the presence of the pump and the noise and vibration associated with its function could interfere with the stress response, the potential effects of these components were evaluated. The following three experimental groups were tested in triplicate: no pump, pump off and pump on. Each group consisted of six glass aquaria containing six zebrafish (36 fish total). Fish were habituated to aquaria for at least three days; after this period, they were sampled to measure whole-body cortisol levels.

### Procedures and Techniques - Cortisol Extraction and Analysis

Tissue cortisol levels were used as an indicator of stress response. Fish were captured and immediately frozen in liquid nitrogen for 10–30 s, followed by storage at −20°C until cortisol extraction. To prevent any handling-induced stress response, the time period between their capture and killing was <30 s.

Whole-body cortisol was extracted using the method described by Sink et al. [26]. Each fish was weighed, minced and placed in a disposable stomacher bag with 2 mL phosphate buffered saline (PBS, pH 7.4) for 6 min. The contents were then transferred to a 10-mL screw top disposable test tube, to which 5 mL of laboratory grade ethyl ether was added. The tube was vortexed for 1 min and centrifuged for 10 min at 3000 rpm, after which the sample was immediately frozen in liquid nitrogen. The unfrozen portion (ethyl ether containing cortisol) was decanted and transferred to a new tube and completely evaporated under a gentle stream of nitrogen for 2 h, yielding a lipid extract containing the cortisol, which was stored at −20°C.

The accuracy of cortisol detection was tested by calculating the recoveries from samples spiked with known amounts of cortisol (50, 25 and 12.5 ng mL^−1^). The mean detection accuracy of spiked samples was 94.3%. All of the cortisol values were adjusted for recovery with the following equation: cortisol value = measured value×1.0604.

Tissue extracts were re-suspended in 1 mL PBS, and whole-body cortisol levels were measured in duplicate samples of each extract using a commercially available enzyme-linked immunosorbent assay kit (EIAgen™CORTISOL test, BioChem ImmunoSystems). This kit was fully validated for *Nile tilapia* tissue extracts using methodology proposed by Sink et al. [26]. Precision was tested by performing 12 repeated assays on seven randomly chosen samples on the same plate and calculating the intra-assay coefficient of variation (CV). Reproducibility was tested by assaying the same samples on different plates and calculating the inter-assay CV. To test for linearity and parallelism, the tissue extracts underwent serial dilutions in the buffer provided with the kit. A strong positive correlation between the curves was observed (*R* ^2^ = 0.8918), and the samples had low inter- and intra-assay CV values (7–10% and 5–9%, respectively).

### Statistical Analysis

Hartley’s tests and Kolmogorov–Smirnov tests were used to determine the homogeneity of variance and normality, respectively. Log-transformation was carried out when necessary. Because the standards for using parametric tests were met, an analysis of variance complemented by Tukey’s multiple range test was performed, and this analysis was used to compare all of means. Difference were considered statistically significant when P<0.05.

## Results

### Study 1 - Alcohol Concentration of 0.25%

**Figure 3:**
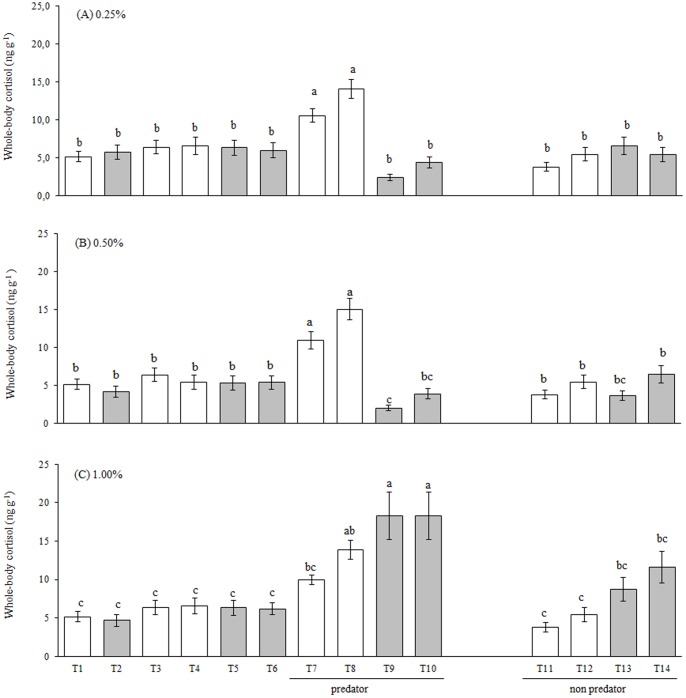
Whole-body cortisol concentrations measured in fish from different experimental groups. Animals were exposed to 0.25% (A), 0.50% (B) or 1.00% (C) alcohol. The values are expressed as the mean ± standard error of mean. The different small letters after each mean indicate significant differences (P<0.0001, ANOVA, followed by Tukey’s multiple range test, see details in the text). White and gray bars represent treatments without or with alcohol, respectively. See the Fig. 2 for treatment descriptions.

An ANOVA indicated differences between the groups (F_13,56_ = 9.825, P<0.0001, Fig. 3A). Control DF (T3) and RF (T4) without predator SF presented low basal values of whole-body cortisol. Alcohol exposure (RF, T5 and DF, T6) did not affect these levels under these conditions. In the situations without stimuli fish (T3 to T6), both DF and RF fish presented similar levels of whole-body cortisol. The presence of the predatory SF significantly increased cortisol levels in both the DF and RF groups (T7 and T8). Exposure to 0.25% alcohol blocked the stress response in the DF and RF because the observed cortisol levels were similar those from fish exposed to basal conditions (T9 and T10). In the presence of the non-predatory fish, the cortisol levels were similar to the control values in the DF and RF groups, regardless of alcohol exposure (T11 to T14).

### Study 1 - Alcohol Concentration of 0.50%

An ANOVA indicated differences between the groups (F_13,56_ = 13.776, P<0.0001, Fig. 3B). Control DF (T3) and RF (T4) without predator stimuli fish presented lower basal values of whole-body cortisol. Alcohol exposure did not affect these cortisol concentrations under these conditions. As verified in the experiment with alcohol concentration of 0.25%, in the situations without stimuli fish (T3 to T6), both DF and RF fish presented similar levels of whole-body cortisol. Similarly to the previous experiment, the presence of a predatory fish significantly increased cortisol levels in both the DF and RF groups (T7 and T8). Exposure to 0.50% alcohol blocked the stress response in the DF and RF, as suggested by cortisol levels that were similar or lower to the basal condition (T9 and T10). In the presence of the non-predatory fish, the cortisol levels were similar to the control values in the DF and RF groups, regardless of alcohol exposure (T11 to T14). None of the comparisons, by Student’s “t” test, between the means DF and RF fish in each condition showed statistically significant differences.

### Study 1 - Alcohol Concentration of 1.00%

An ANOVA indicated differences between the groups (F_13,56_ = 9.878, P<0.0001. Fig. 3C). Control DF and RF without predator SF presented low basal concentrations of whole-body cortisol. Alcohol exposure did not affect cortisol concentrations. As verified in the other experiments fish from situations without stimuli (T3 to T6), both DF and RF fish presented similar levels of whole-body cortisol. The presence of the predatory fish significantly increased cortisol levels in the RF group (T8) in relation to basal situations (T1 and T2). Contrary to the lower alcohol concentrations, exposure to 1.00% alcohol potentiates the stress response in the DF and RF groups (T9 and T10), as the cortisol levels were higher than the control values. None of the comparisons, by Student’s “t” test, between the means DF and RF fish in each condition showed statistically significant differences.

### Study 2 - Effect of Alcohol on Stress Response

**Figure 4:**
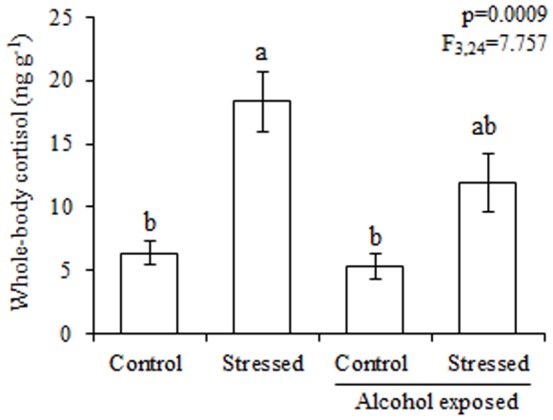
Whole-body cortisol measured in control and stressed fish that were either untreated or exposed to 0.50% alcohol. The values are expressed as the mean ± standard error of mean. Different letters above the histograms indicate significant differences between means (ANOVA, followed by Tukey’s multiple range test, see details in the text).

An ANOVA indicated differences between the groups (F_3,24_ = 7.1, P = 0.0009, Fig. 4). As expected, acute stress significantly increased the cortisol levels; however, exposure to 0.50% alcohol attenuated the magnitude of whole-body cortisol after acute stress.

### Study 3 - Effect of the Submersible Pump

**Figure 5:**
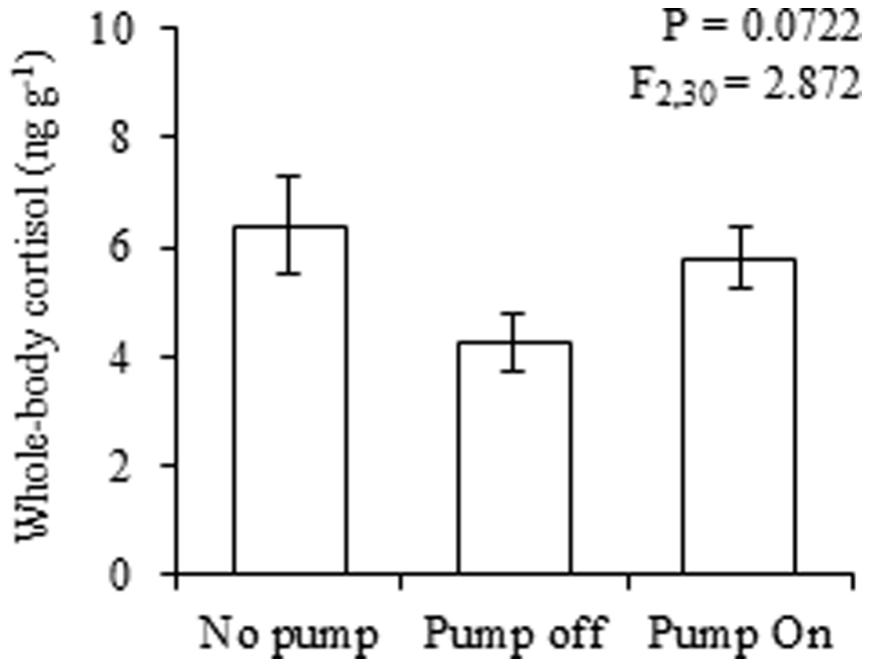
Effect of the presence or absence of the submersible pump on zebrafish cortisol levels. The values are expressed as the mean ± standard error of mean. No significant differences were observed between the experimental groups (ANOVA, followed by Tukey’s multiple range test, see details in the text).

The presence of the submersible pump has no effect on the resting whole-body cortisol levels (Fig. 5). The cortisol levels in fish in the presence of the pump was similar to control fish without a pump, regardless of whether the pump was turned off or on (F_2,30_ = 2.9, P = 0.0722).

## Discussion

We demonstrated that visual contact with a predator triggers activation of the stress axis in zebrafish (T7). We also observed the typical stress response in zebrafish receiving water (RF) from conspecifics after visual contact with a predator (DF), indicating that DF fish chemically communicate the predation risk to RF (T8). These results are in agreement with previous studies investigating both the stress response triggered by visual contact with a predator [12], [27] and the chemical communication of predation risk [10] and/or handling stress [9].

Despite the fact that the Tiger Oscar is an allopatric predator in relation to zebrafish, we previously show that *D. rerio* could recognize certain fish characteristic and behaviors that indicated Tiger Oscar as possible predator. Also, zebrafish did not recognize goldfish as a possible predator [12], [13]. The present study confirms these situations based on data from T7 and T11 groups, validating the use of these species as a predator and non-predator stimuli fish.

We show, for the first time, that exposure to low alcohol concentrations impairs stress axis activation (i.e. values similar or lower than those measured in fish from control situations T1 and T2) in fish visually exposed to a predator (DF, T9) and also in fish receiving water (RF, T10) from the visually exposed fish.

Because the response to stress challenges was not blocked by alcohol exposure in the study 2 (physical stress), we hypothesized that alcohol interferes with the axis by decreasing the danger perception of DF and subsequently reducing the release of the chemical disturbance substance to communicate this imminent threat to RF. This hypothesis is consistent with previous studies in which acute alcohol exposure decreased fear reactions in zebrafish [1], [27] and avoidance behavior to a predator [2], [27]. Additionally, ethanol was described as an anxiolytic-like substance that reduces shoal cohesion in zebrafish [3], [15]. The specific mechanism related to these effects is unclear but is likely mediated by GABA_A_ receptors [28], [29]. GABA_A_ receptors may be involved in HPA axis modulation because the increase in ethanol consumption was associated with reduced blood corticosterone levels, which is indicative of a dampened HPA activation and can be reversed by administration of the GABA_A_ receptor antagonist picrotoxin into the paraventricular nucleus in rats [30].

At alcohol concentrations of 0.25 and 0.50%, our hypothesis that alcohol reduces the perception of the predation risk by the DF, which consequently prevents communication of this imminent risk to RF, is reinforced by the treatments in the presence of a non-predatory fish that did not elicit any hormonal response in both DF and RF. Both the DF and RF groups (T11 to T14) had no reaction in the presence of a large *C. auratus*.

The absence of differences between DF and RF fish without stimuli fish (T3 to T6) also reinforce our main hypothesis.

A possible effect of alcohol on the capacity of the adrenal gland to synthetize cortisol was ruled out based on the results of Study 2, which shows no direct effect of ethanol exposure on stress reactivity. However, because both DF and RF were exposed to alcohol, the effect of alcohol on the ability of the RF to perceive the substance released by the DF cannot be discarded.

Interestingly, treatment with 1.0% alcohol showed different results compared with treatment with the lower alcohol concentrations. At this higher concentration, both the DF and RF groups had increased cortisol levels in the presence of a predator. The alcohol exposure appears to potentiate the risk perception and chemical communication of this predation risk. These results were unexpected because alcohol has anxiolytic and central nervous system depressor effects [1], [2], [15]. Despite this U-shaped response in this exact dose range was already know, at least behaviorally [1] (i.e. 0.25 and 0.50% dosages induces hyperactivity that shifts to hypoactivity at 1.00%), for hormonal concentrations there is still no explained.

The augmented reactivity induced by the higher alcohol concentration is reinforced by the significant cortisol elevation in fish receiving (T14) water from the DF in contact with a non-predator (T13). The cortisol levels were not significantly elevated in the DF group, suggesting that the chemical communication of a perceived threat does not require full HPI axis activation. The nature of the molecules used for this communication has yet to be elucidated.

This study shows that visual contact with a predator triggers the stress axis activation in zebrafish and that fish chemically communicate predation risk, as indicated by the stress response in zebrafish receiving water from these conspecifics. The novelty of this work is that exposure to low concentrations of alcohol blocks both stress axis activation and chemical communication of predation risk. Our primary hypothesis is that alcohol interferes with the perception of danger by DF fish and the consequent release of the chemical disturbance substance to communicate this imminent threat to RF.

Despite the yet unknown mechanism underlying this impaired risk perception in zebrafish, we can trace a parallel with the effects of alcohol in humans, since people exposed to alcohol present an impaired ability to discriminate threatening from non-threatening cues [31], [32]. Together with other research results and with the translational relevance of this fish species, our data points to zebrafish as a promising animal model to study alcoholism.
